# Optimized Active Noise Cancellation for Hearing Tests Using Auditory Masking Characteristics

**DOI:** 10.1109/JTEHM.2025.3629999

**Published:** 2025-11-06

**Authors:** Hsiu-Lien Cheng, Ying-Hui Lai, Po-Hsun Huang, Wen-Huei Liao

**Affiliations:** Department of Otolaryngology-Head and Neck SurgeryTaipei Veterans General Hospital Taipei Taiwan; Department of Biomedical EngineeringNational Yang Ming Chiao Tung University Taipei Taiwan; Ph.D. Program in Regulatory Science and PolicyNational Yang Ming Chiao Tung University Taipei Taiwan

**Keywords:** Active noise cancellation, hearing test, noise control, noisy environment

## Abstract

Objective: Environmental noise poses a major barrier to the accuracy of self-administered hearing tests conducted outside clinical settings. There is a pressing need for effective noise control solutions to enable reliable hearing threshold measurements in everyday environments. This study introduces an optimized active noise cancellation (ANC) technique based on auditory masking characteristics. Method: The method was implemented in a mobile hearing test system using calibrated true wireless Bluetooth earphones. Electroacoustic validation and clinical testing were conducted across four ANC scenarios: normal, generic ANC off, generic ANC on, and optimized ANC on in 65 dB(A) pink noise. Results: A total of 50 participants completed hearing tests at eight frequencies (0.25–8 kHz), and results were compared to standard audiometry. The optimized ANC yielded the highest signal-to-noise ratio in noisy conditions and demonstrated strong agreement with standard hearing thresholds (r = 0.99, p <.01) in normal environments. Under 65 dB(A) noise, the proposed method significantly outperformed generic ANC with smaller hearing measurement error, improving threshold accuracy across most frequencies. Conclusion: The proposed ANC technique enhances hearing test reliability in noisy conditions, supporting accurate, self-administered hearing assessments outside clinical settings. This technology has strong potential for home or community-based hearing healthcare applications.

## I. Introduction

Hearing tests have become crucial components of auditory healthcare [1], [2]. Consequently, simplified hearing test devices have been progressively developed in recent years [3], [4], [5], [6], [7], [8]. Reportedly, these devices can achieve results consistent with those obtained from standard hearing test rooms in quiet environments at approximately 40 dB(A). However, there remains room for improvement under noisy conditions [9], [10]. In other words, the background noise remains a major obstacle to the widespread adoption of hearing test tools.

The previous studies showed that the background noise affects hearing tests primarily because when the frequency of environmental noise is close to that of the test signal, two sounds with similar frequencies can interfere with each other in terms of the traveling wave amplitude on the cochlear basilar membrane [11], [12], [13], resulting in a masking effect [14], [15]. Particularly, the critical bandwidth of the noise center frequency is closely related to the pure-tone hearing threshold [15], [16]. The closer the noise center frequency is to the pure-tone frequency, the more noise leaking into the ear canal masks the pure-tone and causes an increase in the threshold (leading to poorer hearing). Therefore, noise control in hearing test environments is essential to ensuring the accuracy of hearing measurements.

There are two typical methods of eliminating environmental noise leakage into the ear canal: passive and active noise cancellation (ANC). Passive noise cancellation uses physical properties to block noise; however, its effectiveness can be reduced by factors, such as device size, fit, or material [17], [18], [19]. In contrast, ANC uses sensors to detect environmental noise and algorithms to calculate filter parameters, allowing the earphone speaker to generate an inverse sound wave in the ear canal and create destructive interference to reduce noise [20]. Modern ANC systems, particularly the hybrid architectures used in consumer earphones, rely on both external (feed-forward) and internal (feed-back) microphones to accurately sample the noise field. The effectiveness of these systems is closely linked to the physical fit of the earphone, as a proper acoustic seal is crucial for both passive noise isolation and the creation of a stable environment for the ANC algorithm to operate. While significant variations in earphone position relative to a noise source can alter the captured sound field, these systems are generally designed to be robust against minor positional shifts typical of everyday use, ensuring consistent performance once tuned to the device’s specific mechanical and acoustic properties. In 2008, Bromwich applied ANC headphones to hearing tests [21] and demonstrated that, regardless of the noise volume, hearing below 1 kHz was affected. The results confirmed that the ANC headphones considerably reduced the threshold shifts caused by noise. However, while these ANC headphones could significantly improve hearing thresholds in a 40 dB sound pressure level (SPL) noise environment, they achieved results consistent with standard hearing tests only in a 30 dB SPL noise environment. Therefore, optimizing the noise-canceling technology is necessary to improve the accuracy of hearing tests using ANC headphones. In 2017, Saliba et al. designed two hearing test applications paired with ANC headphones and validated the system in noisy environments. The results showed that the performance was similar to that of a standard hearing test room in 50 dB white noise [22]. In 2022, Cheng et al. explored the correlation between pure-tone signals under different noise levels using commercial ANC headphones [23] and compared them with quiet conditions to verify the accuracy of hearing screening [24]. The study showed that commercial ANC headphones maintained good signal characteristics (above 95%) for hearing screening accuracy in 50 dB SPL pink noise; however, the accuracy decreased at 60 dB SPL. Notably, the average noise level in everyday environments is typically 60–65 dB(A) [25]. Therefore, the use of commercial ANC products for hearing testing in real-world environments can be improved.

Recently, ANC technology has been applied to True Wireless Stereo (TWS) headphones. These headphones use fast chip processing [26], [27], [28], [29], [30] to quickly generate anti-noise signals [31], [32], [33], [34], thereby reducing the impact of environmental noise leakage into the ear canal. However, the optimized ANC parameters must be adjusted according to the acoustic characteristics of the ear canal for a more precise application [35]; meanwhile, the masking effect of cochlear physiology should be considered. Therefore, this study aimed to design the necessary noise-canceling parameters for ANC headphones based on an auditory masking phenomenon, called “optimized ANC,” to achieve a deep noise reduction around the center frequency and bandwidth defined by the ANSI narrowband noise standard for hearing test applications. Furthermore, clinical experiments validated the effectiveness of the optimized ANC method in hearing test applications compared with that of the baseline ANC system in this study. Our objective was to create an optimized ANC design that could facilitate the development of a reliable home-based hearing screening tool that would benefit users in the gain selection of over-the-counter hearing aids.

## II. Methods

### A. Proposed System

This cross-sectional cohort study evaluated an optimized noise reduction technique, developed based on auditory masking’s physiological characteristics, by comparing its performance with a generic ANC approach. The generic ANC system refers to the built-in, factory-default noise cancellation feature of the commercial GMI Slide2 TWS earphones. This system is designed as a one-size-fits-all solution for everyday noise environments and is not specifically optimized for the discrete frequencies used in audiometric testing.

Previous research has indicated a close relationship between the critical bandwidth of the noise center frequency and auditory masking effects [15], [36]. Masking implies that the threshold of one sound (signal) is raised by the presence of another sound (masker). Masking characteristics reflect the displacement patterns along the basilar membrane caused by two sounds with different frequency components. The strongest masking magnitude occurs around the masker frequency, and the amount of masking decreases with distance from this masker frequency. Once the masker frequency exceeds a certain bandwidth, the signal threshold will not change significantly, which is the critical bandwidth for the masker. Therefore, the American National Standards Institute (ANSI) has established standard hearing test procedures that use narrowband noise as a masker, with the narrowband noise bandwidth determined based on the hearing test frequency to eliminate cross-hearing [37]. This viewpoint gives rise to the idea of designing the ANC filter parameters such that the acoustic components of the critical bandwidth centered on the pure-tone frequency will be eliminated to enhance the pure-tone signal-to-noise ratio (SNR) and achieve a higher hearing test measurement accuracy outside the booth.

**FIGURE 1.**
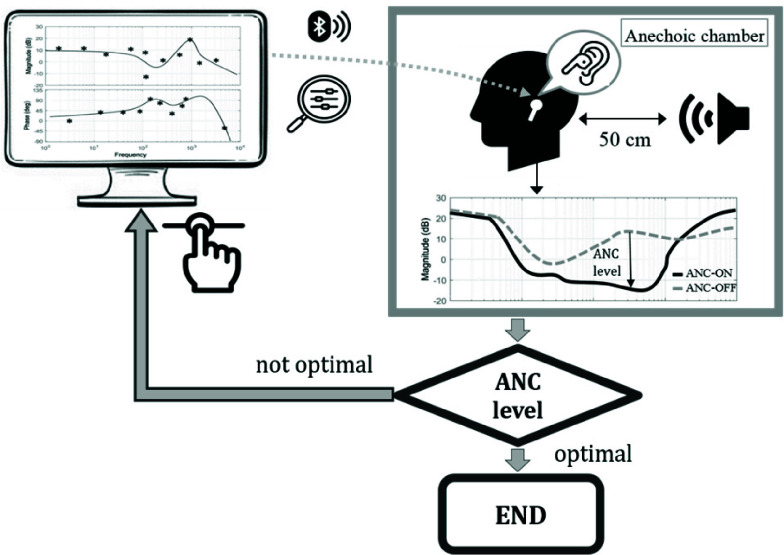
The design flowchart of the proposed optimized active noise cancellation (ANC) system, denoted optimized ANC in this study. It targets the frequency range of narrowband masking noise to optimize ANC technology. Through parameter adjustments in the chip system, each target frequency band can achieve the best noise reduction performance.

Fig. 1 illustrates the proposed ANC optimization method, denoted as “optimized ANC” in this study. We set the narrowband noise bandwidth, as defined by the ANSI standard (Appendix Table 1) (see the Supplementary Material), as the optimization target for the ANC technology. Using the ANC design tool of the Bluetooth chip, we tuned the biquad filter of the ANC feedforward architecture. The filter parameter adjustments consider the magnitude and phase characteristics of the earphone mechanism. Therefore, the target frequency response curve achieves better ANC anti-noise depth, and the adjusted ANC filter parameter reaches an optimized active noise reduction in the target frequency range, improving the anti-noise efficiency of the system under noisy conditions. Subsequently, electroacoustic measurements were used to repeatedly confirm that the filter parameters achieved the optimized noise reduction depth within this narrowband noise bandwidth (with a maximum reduction of 40 dB), selecting the filter coefficients that provided the most substantial noise reduction.

**TABLE 1:** Descriptive Statistics of the Participants (n = 50)

Variable	n (%)/Age (Mean ± SD, Min, Max)
Sex/Age	
Male, n (%) / Age	23 (46%)/40.0 ± 16.5 (20, 72)
Female, n (%) / Age	27 (54%)/41.9 ± 13.7 (22, 73)
Average Hearing Threshold (dB HL) #	
Left Ear	18.5 ± 16.1
Right Ear	17.8 ± 16.5

Note (#): The average hearing threshold is calculated as the mean of thresholds at 0.5, 1, and 2 kHz. HL, hearing level

### B. Experimental Equipment for Pure-Tone Accuracy Calibration

The experimental equipment in this study comprises two main parts: (a) TWS earphones and (b) an Android-based hearing test application (Hearing Test APP). The GMI Slide2 model (GMI Slide2; Realtek RTL8773CO chipset; Bluetooth 5.3) was used for the TWS earphones, allowing the ANC parameters and hearing test volume control to be adjusted using the chip configuration software. All stimuli were streamed over Bluetooth using the Advanced Audio Distribution Profile (A2DP), and the TWS earphones used the mandatory A2DP Subband Codec (SBC) for all electroacoustic and clinical measurements in this study, ensuring reproducibility of the wireless audio path. The second part involved the Hearing Test APP, which was run on a mobile device. To ensure that the output volume of the TWS earphones paired with the Hearing Test APP matched the sound pressure level standards referenced by pure-tone audiometry threshold values, the output of the TWS earphones was measured using a head and torso simulator (45BC-10 KEMAR Head & Torso Simulator, GRAS Sound & Vibration, Denmark, referred to as KEMAR hereafter). Further measurements were conducted to compare the acoustic equivalency with that of an ANSI S3.6 standard audiometer (Audio Star Pro) [37], which uses IP30 insert earphones (RADIOEAR) (Figure 1).

## III. Experimental Design

### A. Objective Electroacoustic Characteristic Verification

To assess the pure-tone SNR and quality of the ANC earphones under 65 dB(A) pink noise, an objective analysis was performed before conducting the clinical experiments. This was done to evaluate the performance of optimized versus non-optimized (i.e., generic) ANC technologies when applied to pure-tones from 0.25 to 8 kHz at a threshold of 25 dB hearing level (HL) in a noisy environment.

The experiment was conducted in a soundproof room using equipment that included the Hearing Test APP, TWS earphones, KEMAR head and torso simulator, speaker, and CLIO electroacoustic analysis software. Before electroacoustic measurements, we explicitly fixed the over-the-air codec to SBC throughout the experiments to avoid variability across host devices and ensure that the same compression scheme was applied across sessions. The experimental procedure involved adjusting the speaker to be parallel to the KEMAR artificial ear at a fixed distance of 50 cm. The microphone in the ear canal was calibrated to a sensitivity of 94 dB SPL at 1 kHz. After verifying that there was no feedback leakage when using the ANC design tool, the speaker played a pink noise. A sound level meter (Center 320, CENTER TECHNOLOGY Corp.) was placed at the KEMAR ear canal opening to measure sound pressure values (in dB(A)) using the fast-sampling mode and frequency-weighted mode, ensuring a 65 dB(A) pink noise environment.

**FIGURE 2.**
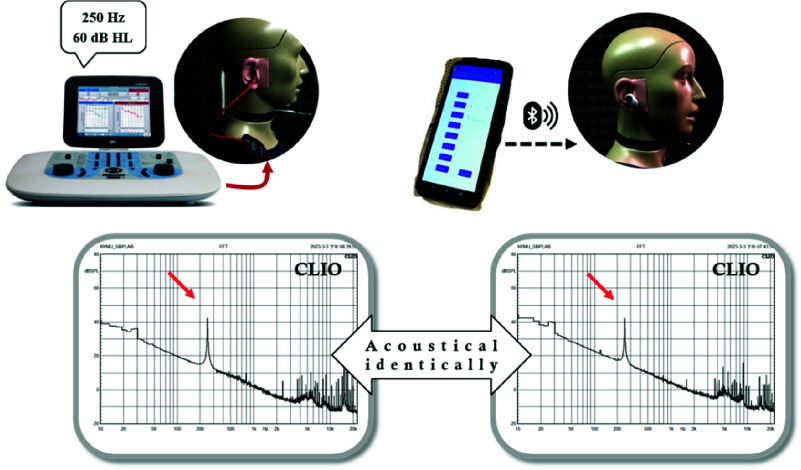
Comparison of acoustic output fidelity. The Fast Fourier Transform (FFT) analysis shows that the 250 Hz pure-tone signal from our proposed wireless system (right) is acoustically identical to that from a clinical audiometer (left), with no evidence of codec-induced artifacts.

Electroacoustic measurements of pure-tone SNRs and quality were then conducted for the following four ANC usage scenarios: Normal environment with ANC turned on (Scenario S1), Noisy environment with ANC turned off (Scenario S2), Noisy environment with generic ANC turned on (Scenario S3), Noisy environment with optimized ANC turned on (Scenario S4). Freedom from codec-related artifacts in the pure-tone stimuli was verified by inspecting the spectrum (FFT) of the acoustic output on a head-and-torso simulator (Figure 2). The optimized ANC method refers specifically to the active noise cancellation technique proposed in this study, which optimizes the ANC parameters based on auditory masking characteristics (see Figure 1 for detailed design flowchart). Finally, the SNR improvement of each method was quantified using the SNR difference calculation (1), where f represents the center frequency of the SNR.

\begin{align*} & {\mathrm {SNR}}_{f} \\ & =\left ({{ {\mathrm {dB ~SPL}}_{\mathrm {25 ~dB ~acoustic~equivalency}} }}\right)-\left ({{ {\mathrm {dB ~SPL}}_{\mathrm {octave~band}} }}\right) \tag {1}\end{align*}

### B. Clinical Hearing Measurement Validation

The hearing test equipment used in this study, including the Hearing Test APP and TWS earphones. The mobile hearing test application follows the Hughson–Westlake down-10/up-5 method [38], [39] to measure hearing thresholds, with familiarization practice performed before threshold testing at each frequency. The threshold was defined as the lowest level at which responses were detected at least 50% of the time using an ascending technique [39]. The Hearing Test APP automates the entire process, including pure-tone playback, frequency changes, and threshold determination. The app was first connected to the TWS earphones via Bluetooth, during which the phone volume was maximized and locked to prevent accidental changes that could affect the test results.

During the familiarization phase, the test began at 1 kHz with a starting level of 30 dB HL. If a response was detected, the system reduced the level by 10 dB; if no response was detected, the system increased the level by 20 dB. Once the response was reversed, threshold measurement began. Based on the responses of the participants, the system automatically adjusted the level by increasing it by 5 dB or decreasing it by 10 dB. Each pure-tone lasted 500 ms, and the participants had up to 2 s to respond. The interval between the tone presentations varied randomly between 600 and 1200 ms. The threshold measurement at 1 kHz was completed once the defined threshold was reached, after which the test automatically proceeded through 2, 3, 4, 6, 8, 0.5, and 0.25 kHz. When using the optimized ANC earphones, the system automatically applied the optimized ANC parameters to each corresponding test frequency. For example, during the 1 kHz test, the ANC parameters for a 1 kHz center frequency were applied. Similarly, during the 0.5 kHz test, the parameters for a 0.5 kHz center frequency were used. Once the hearing tests for all eight frequencies were completed, an audiogram was automatically generated on the mobile screen to display the test results.

**FIGURE 3.**
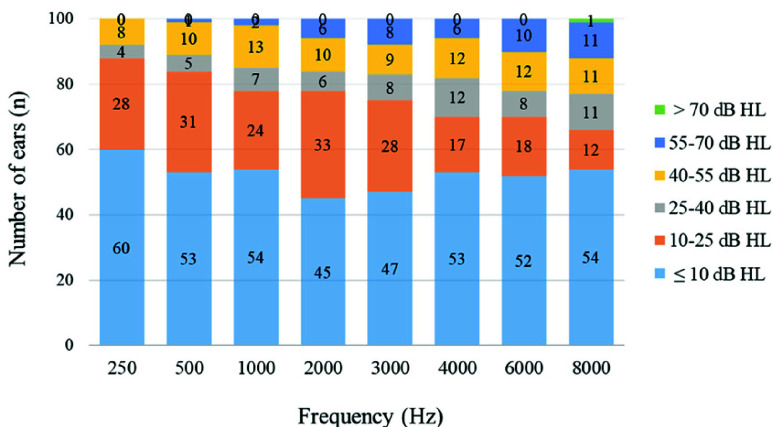
Number of ears categorized by dB hearing level (dB HL). Categorization was as follows: better than 10 dB HL, 10–25 dB HL, 25–40 dB HL, 40–55 dB HL, and 55–70 dB HL and worse than 70 dB HL at 250 to 8000 Hz.

Based on the above procedure and a previous pilot study for evaluating the effectiveness of ANC technology in hearing screening [24], we invited 50 participants to undergo hearing tests in the standard audiometric room of the Otolaryngology Department at the hospital between May and December 2023. The criteria for recruitment included age 20 years and above, with pure-tone average within 60 dB HL. The difference in hearing threshold at any frequency should not exceed 40 dB between two ears in case of cross-hearing. Individuals with an air-bone gap exceeding 40 dB between both ears, physical activity restriction, attention deficit, or difficulty in understanding the experimental procedure were excluded from the study. The participant information is summarized in Table 1. A histogram of the HL across the participants is presented in Fig. 3.

**FIGURE 4.**
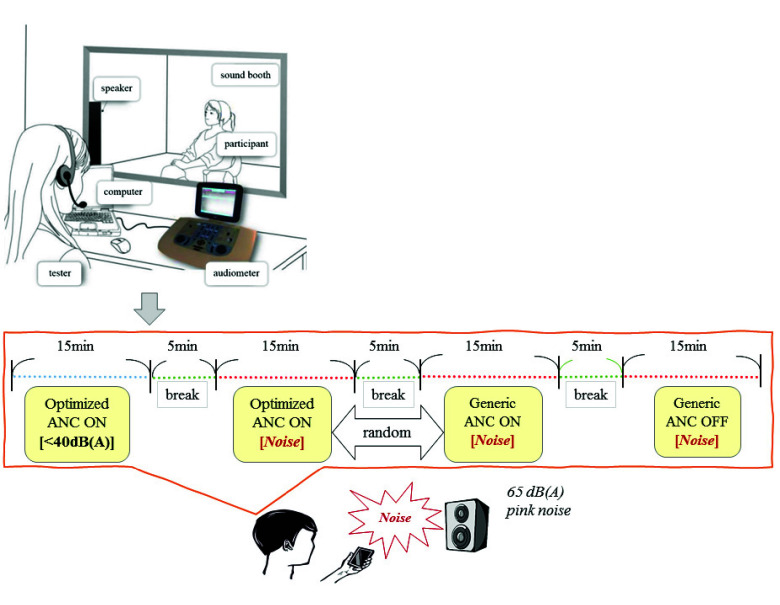
Flowchart of the clinical hearing test experiment, (Top) Standard hearing test, (Bottom) Active noise cancellation (ANC) hearing test.

Fig. 4 illustrates the clinical study workflow, which included a baseline standard hearing test, followed by hearing tests under the four ANC conditions using the experimental equipment. A standard hearing test (GSI Audiostar Pro with RadioEar IP30 insert earphones) was used to screen the participants. Individuals with average hearing thresholds above 65 dB HL or an air-bone gap exceeding 40 dB between the ears were excluded from the study. Only participants who consented to the study after receiving a thorough explanation of the research methods and signed an informed consent form were included in the tests. To minimize potential errors when operating the mobile Hearing Test APP, Scenario S1 (normal environment with ANC on) was conducted first. To avoid discomfort caused by noise, Scenario S2 (noisy environment with ANC off) was conducted last. Scenarios S3 (generic ANC on) and S4 (optimized ANC on) were randomly administered to control participant fatigue. All data, including participant information, standard hearing thresholds, and hearing thresholds from scenarios S1–S4, were recorded on an Excel worksheet for analysis. This clinical study was approved by the Institutional Review Board of the hospital.

### C. Statistical Analysis Methods

The study data for included one set of standard hearing thresholds and four sets of hearing measurements under ANC conditions. Data validation was performed before statistical analysis. The study enrolled 50 participants. One participant did not complete the most challenging scenario (S2: noisy, ANC off), and 16 data points in S2 (1 participant $\times 2$ ears $\times 8$ frequencies $\times 1$ scenario) were treated as missing data. The 26 outlier data points resulting from false responses were removed from the study data. Finally, the study datasets were adjusted for data consistency considering the limitations of the TWS earphone drivers, which may not consistently maintain sound pressure levels below 25 dB HL. For data consistency, a hearing threshold of 25 dB HL was set as the standard for normal hearing. For any threshold below 25 dB HL, the data were categorized to 25 dB HL. If the threshold exceeded 25 dB HL, the original value was retained. Any instances where hearing measurements under ANC conditions improved by more than 15 dB compared with the standard threshold were treated as false responses, and those data points were excluded from the statistical analysis. For example, if the left ear threshold at 250 Hz was 10 dB HL after processing, it was categorized to 25 dB HL. If the threshold of the right ear at 250 Hz was 35 dB HL, then the value remained at 35 dB HL. If the standard threshold for the right ear at 250 Hz was 35 dB HL and the ANC test result was 20 dB HL, the data point was excluded from the analysis. After processing, the experimental data were analyzed by using IBM SPSS software to compute the mean absolute error (MAE) and perform a Pearson correlation analysis [40], [41] in order to investigate the mean measurement error and the correlation between the optimized ANC technology and standard pure-tone hearing thresholds. Finally, a simple linear regression was used to examine the predictive relationship between ANC scenarios and the standard audiometric HL. The significance level was set at $\alpha < 0.05$, and for two-tailed tests where the p-value was $\le \alpha $, the correlation (r) was considered statistically significant.

## IV. Results

### A. Earphone Calibration Results

Table 2 presents the calibration coefficients of the TWS earphones used in this study and the corresponding equivalent SPL for pure-tone outputs. The experimental results showed that after calibration, the equivalent SPL of the left and right TWS earphones at 60 dB HL across various frequencies differed by <1.0 dB compared with those of the standard audiometric insert earphones, except for the right generic TWS earphones at 8 kHz, where the difference was slightly higher at 1.7 dB. For all other frequencies, the differences were below 3.0 dB, thus meeting the ANSI S3.6 standard. To minimize the calibration bias, TWS earphone calibration was performed with the output of the insert earphones as the target for the equivalent SPL. The results show that although the calibration coefficients of the generic and optimized TWS earphones are not exactly the same, their structural designs are identical. Therefore, the calibration coefficients between the left and right earphones (between-class) and within the same earphone (interclass) were similar. Table 3 records the linearity check of the pure-tone output from the hearing test equipment, showing that the average error of the linear output measured by KEMAR for the TWS earphones is within ± 1 dB, which complies with the ANSI S3.6 standard.

**TABLE 2:** Equivalent Sound Pressure Levels of TWS Earphones at 60 dB HL and Their Calibration Coefficients

Ear	Earphones		Frequency (Hz)							
			250	500	1k	2k	3k	4k	6k	8k
Left ear	Insert earphone#	Calibration coefficient	77.1	69.1	65.1	71.6	73.9	70.7	75.3	73.6
	generic TWS		76.4	69.9	65.3	72.8	73.6	71.6	75.3	73.7
			0.043	0.02	0.0095	0.01	0.009	0.0085	0.011	0.018
	optimized TWS	Calibration coefficient	76.3	68.0	63.8	71.0	73.4	70.6	74.5	74.4
			0.105	0.052	0.019	0.023	0.017	0.018	0.027	0.046
Right ear	Insert earphone#	Calibration coefficient	77.8	69.9	65.8	72.3	74.9	72.0	75.3	74.3
	generic TWS		77.0	70.3	66.2	72.3	73.6	72.0	75.9	76.0
			0.051	0.026	0.0145	0.013	0.0105	0.0101	0.013	0.014
	optimized TWS	Calibration coefficient	77.7	69.5	65.3	71.9	74.0	71.0	75.8	75.0
			0.095	0.051	0.029	0.024	0.02	0.02	0.031	0.038

Notes (#): The values in dB SPL for the insert earphones (RadioEar IP30) at 60 dB HL. Data are presented as dB SPL/calibration coefficient, representing the system’s equivalent sound pressure level at 60 dB HL after calibration.

**TABLE 3:** Linearity Check of Pure-Tone Playback by TWS Earphones

Hearing level (dB HL)	Frequency (Hz)								
	Ear	250	500	1k	2k	3k	4k	6k	8k
60	Left	76.3	68.0	63.8	71.0	73.4	70.6	74.5	74.4
	Right	77.7	69.5	65.3	71.9	74.0	71.0	75.8	75.0
55	Left	71.0	62.8	58.7	66.0	68.4	65.6	69.5	69.4
	Right	72.5	64.3	60.2	66.9	69.0	66.0	70.8	69.9
45	Left	60.5	52.6	48.7	56.0	58.4	55.60	59.5	59.4
	Right	61.9	54.0	50.2	56.9	59.0	56.0	60.8	59.9
35	Left	50.2	42.4	38.0	46.0	48.4	45.6	49.5	49.4
	Right	51.7	43.9	40.2	46.9	49.0	46.0	50.8	50.0
25	Left	40.0	32.4	28.6	35.9	38.4	35.5	39.5	39.4
	Right	41.5	33.8	30.1	36.8	39.0	36.0	40.8	40.0

To ensure consistency between the TWS earphone calibration results and clinical hearing test outcomes, 16 participants with mild-to-moderate symmetric hearing loss in both ears were recruited for biological calibration. This calibration aimed to assess whether there were significant differences between the hearing thresholds measured using the experimental equipment calibrated according to Table 4 and those measured using standard audiometric equipment. Table 4 shows the error values from the biological calibration of the TWS earphones, indicating that the average threshold error between the TWS earphones and standard audiometric equipment for frequencies between 0.25 and 8 kHz was within 10 dB. The overall average error across all frequencies was 3.7 dB for the left ear and 2.8 dB for the right ear.

**TABLE 4:** Error Values From Biological Calibration of TWS Earphones (Unit: dB) (n = 16)

TWS Earphone	Frequency (Hz)							
	250	500	1k	2k	3k	4k	6k	8k
Left ear	2.8 ± 4.8	5.3 ± 5.6	5.3 ± 3.4	1.3 ± 3.9	1.9 ± 5.7	0.9 ± 4.6	5.0 ± 4.8	7.2 ± 6.0
Right ear	0.6 ± 5.4	3.8 ± 2.9	3.1 ± 3.6	1.6 ± 4.7	2.5 ± 6.8	-0.3 ± 3.9	3.8 ± 5.3	7.2 ± 9.5

Note: The data is presented as mean ± standard deviation.

### B. Electroacoustic Analysis of Pure-Tone SNR of ANC Technology

**FIGURE 5.**
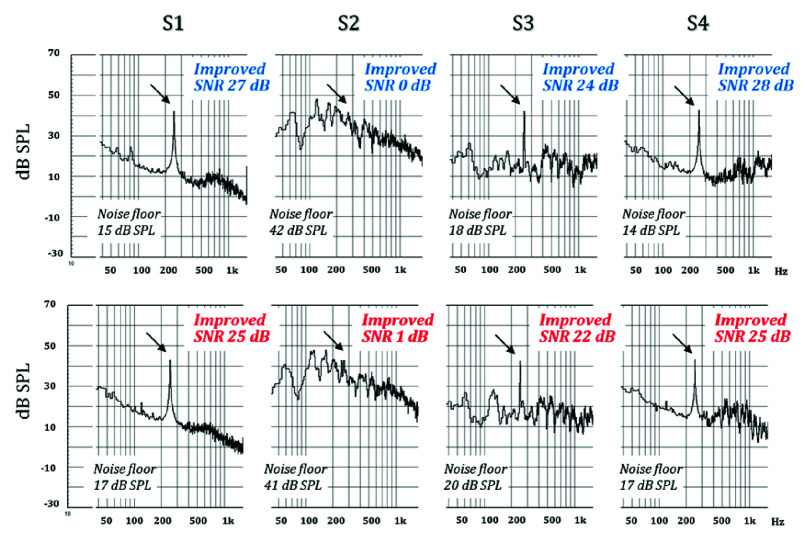
Electroacoustic measurements at 250 Hz under four active noise cancellation (ANC) test conditions. The top row shows the left ear, and the bottom row shows the right ear. The arrows indicate the position of the signal, and the level at 250 Hz was 42 dB SPL for both ears.

**FIGURE 6.**
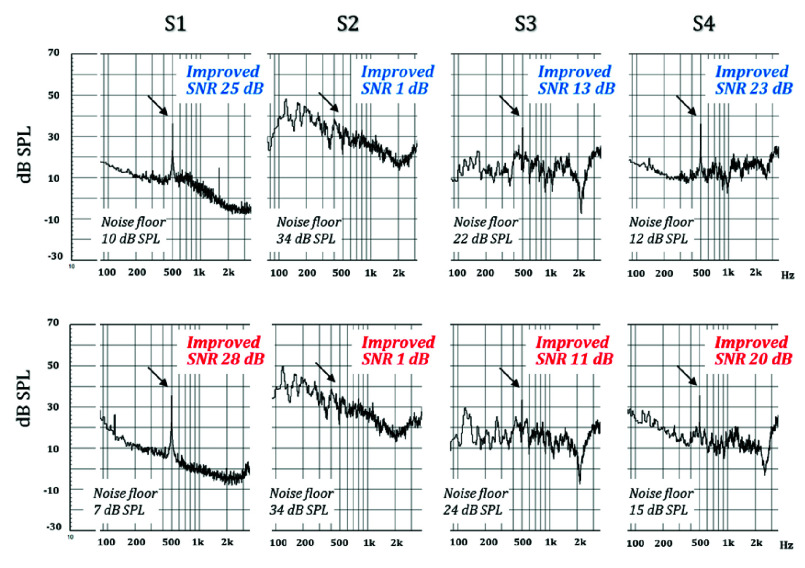
Electroacoustic measurements at 500 Hz under four active noise cancellation (ANC) test conditions. The top row shows the left ear, and the bottom row shows the right ear. The arrows indicate the position of the signal, and the level at 500 Hz was 35 dB SPL for both ears.

**FIGURE 7.**
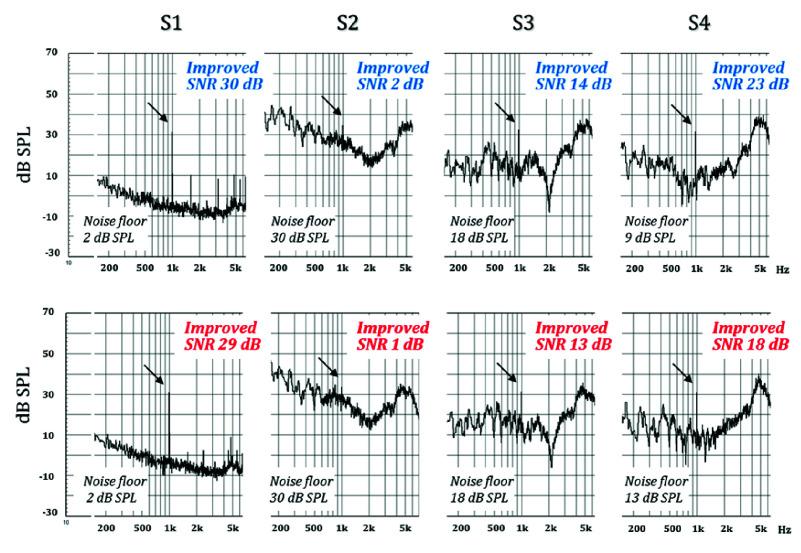
Electroacoustic measurements at 1000 Hz under four active noise cancellation (ANC) test conditions. The top row shows the left ear, and the bottom row shows the right ear. The arrows indicate the position of the signal, and the level at 1000 Hz was 32 dB SPL for the left and 31 dB SPL for the right.

Before the clinical experiment, the pure-tone SNR of the four ANC conditions was objectively analyzed using KEMAR and CLIO to evaluate the performance of the ANC technology. The SNR was calculated using the equivalent SPL of a 25 dB HL pure-tone as the signal strength, while the noise floor was estimated as the average SPL of the octave band centered on the pure-tone frequency (see Equation 1). Fig. 5–7 show the pure-tone SNRs for the four ANC test conditions at 0.25, 0.5, and 1 kHz. Overall, when observing the SNR with the ANC on or off, the pure-tone SNR was higher when the ANC was turned on, with the best performance observed in Scenario S1, where the SNR exceeded 25 dB (ranging from a minimum of 25 dB to a maximum of 30 dB), which was higher than that in Scenarios S2 and S4. Regarding the ANC technology used, the results showed that the SNR in Scenario S4 was higher than that in Scenario S3 at all three frequencies (0.25, 0.5, and 1 kHz). This indicates that optimized ANC technology under noisy conditions results in a higher pure-tone SNR than when using generic ANC.

When evaluating the performance of ANC technology for a pure-tone SNR at 0.25, 0.5, and 1 kHz, the results revealed that both optimized and generic ANC technologies exhibited similar noise cancellation capabilities near 250 Hz while maintaining a consistent noise floor of approximately 20 dB. However, at 0.5 and 1 kHz, optimized ANC showed better noise cancellation performance than generic ANC, as the noise floor at these frequencies was lower when optimized ANC. In addition, the results indicated some discrepancies in the noise cancellation performance of the left and right earphones at 1 kHz when optimized the ANC (S4).

### C. Clinical Performance Results of Optimized ANC Technology

A total of 50 participants (100 ears) were included in this study, with an average age of 41.0 ± 15.1 years. There were 23 men and 27 women, and all participants had symmetrical hearing thresholds in both ears. The average hearing thresholds at three frequencies (0.5, 1, and 2 kHz) for the left and right ears were 18.5 ± 16.1 dB HL and 17.8 ± 16.5 dB HL, respectively. Descriptive statistics of the participants are shown in Table 1, and the means and standard deviations of the absolute error of hearing measurement across ANC test conditions for ears with normal hearing and hearing loss are listed in Table 5.

**TABLE 5:** Mean and Standard Deviation (SD) of Absolute Error of Hearing Measurement Across ANC Scenarios by dB Hearing Level Groups

ANC scenario	Frequency (Hz)							
	250	500	1k	2k	3k	4k	6k	8k
gp=0								
SI	4.1 (3.8)	3.8 (3.9)	3.6 (3.5)	3.0(3.3)	3.6 (5.3)	3.3 (3.0)	5.0 (3.0)	7.0 (5.6)
n ears	11	16	22	22	25	30	29	33
S2	12.0 (8.9)	14.3 (8.5)	16.0 (8.8)	8.8 (8.3)	11.1 (8.7)	12.0 (7.6)	12.3 (7.3)	13.8 (9.9)
n ears	10	14	20	20	23	28	28	32
S3	7.5 (4.0)	9.0 (3.9)	11.7(4.0)	7.3 (3.0)	7.4 (5.0)	9.0 (6.5)	9.1 (4.7)	12.0 (5.7)
n ears	12	15	21	22	25	29	28	33
S4	4.5 (5.7)	5.9 (6.1)	6.1 (4.1)	2.0 (3.0)	4.2 (4.3)	4.3 (4.0)	4.7 (3.8)	6.3 (5.3)
n ears	11	16	22	22	25	28	29	34
gp=l								
S1	0.1 (0.5)	0.0 (0.0)	0.1 (0.6)	0.1 (0.6)	0.1 (0.6)	0.1 (1.2)	0.2 (1.0)	0.4 (1.6)
n ears	88	84	78	78	75	70	69	65
S2	12.3 (7.0)	14.5 (6.9)	20.4 (6.0)	10.2 (6.8)	10.9 (7.1)	19.5 (4.6)	14.1 (4.7)	4.7 (5.0)
n ears	87	83	78	78	75	70	70	66
S3	3.4 (7.0)	8.5 (9.2)	12.6 (9.8)	4.9 (5.8)	8.4 (4.8)	18.5 (6.8)	16.0 (5.9)	6.7 (7.0)
n ears	88	82	78	78	75	70	69	64
S4	0.6 (2.1)	2.6 (5.7)	6.8 (7.2)	1.7 (3.1)	2.7 (3.1)	12.1 (4.6)	12.3 (5.8)	1.1 (2.6)
n ears	88	84	77	78	75	70	70	63

Note. gp = 0: the ear with clinical hearing thresholds at the frequency >25 dB HL

gp = 1: the ear with clinical hearing thresholds at the frequency ≤ 25 dB HL).

Mean absolute Error (MAE) quantified the magnitude errors for all four scenarios by comparing them to the clinical baseline. The overall results across ANC test conditions were as follows: S1 (Normal) = 1.1 dB, S2 (Noise, ANC Off) = 13.2, S3 (Noise, Generic ANC) = 9.6 dB, and S4 (Noise, Proposed ANC) = 4.8 dB. In the sub-clinical population stratified by the 25-dB HL criterion, ears with hearing loss had smaller MAEs at 250–8 kHz in S2 but higher MAEs across S1, S3, and S4 ANC test conditions at 250–2000 Hz than those of ears with normal hearing, except for 1 kHz (Table 5). Comparing the performance of two ANC methods, both groups had a larger difference in hearing level measurement when using the generic ANC method rather than optimized ANC methods. The MAEs obtained from hearing loss ears were over 5 dB at all frequencies. Despite the smaller deviation for ears with normal hearing obtained using the optimized ANC method in 65 dB(A) noise than that obtained using the generic ANC method, the MAEs at 1 kHz and 4–6 kHz were slightly higher. The performance of the proposed ANC method was particularly accurate and clinically significant. An overall absolute error of 4.8 dB indicated that the average difference between the results of the self-administered test using the optimized ANC method in a noisy environment and clinical results was no greater than the typical variation. However, owing to the large measurement error found in the ears with hearing loss, and because the optimized ANC method at 1 kHz exceeds the clinical typical variation, the auditory filtering characteristics of hearing-impaired ears and the proposed ANC technique at 1 kHz require further optimization for more reliable and accurate hearing measurement in noisy environments.

Table 6 shows the Pearson correlation analysis results for the four ANC test conditions and their correlations with standard hearing thresholds. The hearing thresholds measured under the four ANC conditions showed a significant positive correlation with the standard hearing thresholds at 0.25 to 8 kHz (p <.01). This indicated that as the standard hearing threshold improved (lower threshold), the ANC-measured hearing threshold also improved (lower threshold), and vice versa. This suggests that the hearing test results obtained using the ANC technology in the experiment were consistent with those obtained in a standard audiometric environment by professional audiologists. Among the four ANC conditions, Scenario S1 exhibited the highest correlation with the standard thresholds, with all frequencies showing a strong positive correlation (r >0.9). Scenario S4 followed, with slightly lower correlation coefficients than S1, but still showed a significantly high positive correlation across all frequencies (r between 0.8 and 0.9). In Scenarios S2 and S3, the correlation between hearing thresholds at 0.25 to 1 kHz and standard thresholds ranged from moderate to high (r between 0.6 and 0.7), while frequencies above 2 kHz showed a strong positive correlation (r above 0.7). Interestingly, for Scenario S2 (ANC-off), the correlations at 0.5, 1, 4, and 6 kHz were higher than those for Scenario S3 (generic ANC-on).

**TABLE 6:** Pearson Correlation Coefficients Between Hearing Thresholds in the Four ANC Test Conditions and Standard Audiometric Thresholds (n = 100 Ears)

ANC Test Condition		Frequency (Hz)							
		250	500	1k	2k	3k	4k	6k	8k
S1	ANC-on, normal environment	. $967\ast \ast $	. $983\ast \ast $	. $987\ast \ast $	. $986\ast \ast $	. $969\ast \ast $	. $975\ast \ast $	. $978\ast \ast $	. $964\ast \ast $
S2	ANC-off, noise	. $663\ast \ast $	. $695\ast \ast $	. $770\ast \ast $	. $845\ast \ast $	. $810\ast \ast $	. $794\ast \ast $	. $880\ast \ast $	. $905\ast \ast $
S3	Generic ANC-on, noise	. $702\ast \ast $	. $670\ast \ast $	. $731\ast \ast $	. $923\ast \ast $	. $895\ast \ast $	. $710\ast \ast $	. $857\ast \ast $	. $917\ast \ast $
S4	Optimized ANC-on, noise	. $931\ast \ast $	. $825\ast \ast $	. $832\ast \ast $	. $960\ast \ast $	. $954\ast \ast $	. $836\ast \ast $	. $845\ast \ast $	. $964\ast \ast $

Note: ** p <0.01 indicates that the correlation was statistically significant.

The results indicated that hearing measurements using ANC earphones were highly correlated with standard hearing tests. However, in the cases of generic ANC or when the ANC is turned off, the correlation between low-frequency hearing thresholds (0.25–1 kHz) and standard hearing tests is slightly lower than the results obtained by optimized ANC technology. For frequencies above 2 kHz, the correlation between hearing thresholds and standard tests remained high, regardless of the ANC technology used. Overall, the results demonstrate a strong correlation between hearing measurements obtained using ANC earphones and standard hearing tests, indicating that the design of the experimental hearing test equipment and the equivalent SPL calibration method for ANC earphones are feasible. Additionally, this study confirmed that optimizing the ANC technology can maintain a high correlation and low measurement errors with standard hearing thresholds in high-noise environments. Although the noise environment of the study can only partially represent real-world conditions, the noise reduction performance of the optimized ANC technology for various noises can be explored and supports its applicability in daily life. Moreover, the hearing test results at all frequencies were superior to those obtained using generic ANC technology.

From Table 6, we also observed that turning off the ANC at 0.5, 1, 4, and 6 kHz yielded a slightly higher correlation with standard hearing tests than using generic ANC. This suggests that a noise level of 65 dB(A) may exceed the noise reduction capabilities of the generic ANC technology. Consequently, in noisy environments, the performance of the generic ANC was similar to that when the ANC was turned off. At frequencies above 2 kHz, the correlation between the hearing test results and standard hearing tests for all four ANC conditions remained high, with the exception of the 4 kHz result in Scenario S3 (r = 0.710). Most of the high-frequency hearing test results showed correlation coefficients above 0.8, indicating that optimized ANC technology provides hearing test results closer to standard audiometric tests, whether in everyday or noisy environments, than generic ANC or when ANC is not used.

Furthermore, a simple linear regression was calculated to evaluate the extent to which the self-administered hearing test in the ANC scenarios predicted the standard audiometric HL at 250–8000 Hz. Appendix Table 2 (see the Supplementary Material) shows the results of the regression analysis. The regression coefficient (B) found in the four ANC scenarios had a positive relationship with the standard audiometric HL at 250–8000 Hz (p<0.0001), affirming the predictive power of the ANC scenarios on the hearing results. In addition, the coefficient of determination (R^2^) indicated a higher R^2^ value for the S4 scenario than that for the S3 scenario at 250–8000 Hz, except for at 6000 Hz. Overall, the linear regression results indicated that the optimized ANC method’s predictions were more accurate and that they better captured variability than the generic ANC method’s predictions of standard audiometric HLs.

Combining the electroacoustic analysis of pure-tone SNRs and the clinical hearing test results of optimized ANC technology shows that the optimized ANC technology can maintain a pure-tone SNR of 18–23 dB at 0.5 and 1 kHz in a 65 dB(A) noise environment (Figures 5–7). However, this is slightly lower compared to the pure-tone SNR at 250 Hz (25–27 dB). Similarly, the clinical hearing test results indicate that the hearing test performance at 0.5 and 1 kHz is inferior to that at 250 Hz.

## V. Discussions

### A. Evaluation of Earphone Calibration

The output of the left and right for generic and optimized TWS earphones at 60 dB HL across various frequencies were equivalent to those of the standard audiometric insert earphones, except for the right generic TWS earphones at 8 kHz. The difference of output might result from the earphone design [42], insertion depth into the ear canal [43], and sound pressure leakage [44] can alter the acoustic impedance of the ear canal volume, thereby affecting the stability of sound pressure at the eardrum. Moreover, biological calibration of the TWS earphones indicating overall threshold error between the TWS earphones and standard audiometric equipment were within 10 dB. For the accuracy assessment of hearing tests, a significant threshold shift defined according to several criteria [45]. These include the threshold difference at a single frequency, average threshold difference across multiple frequencies, and difference exceeding 10 dB between two adjacent frequencies. In this study, we compared individual frequencies and defined a significant threshold shift as a difference >10 dB. The average error for the left ear at 0.5 and 1 kHz was 5.3 dB, whereas the average error at 8 kHz for both ears was 7.2 dB, which was slightly larger than that of the other frequencies. However, the average error for all other frequencies was within 5 dB, indicating that the hearing test equipment used in this study, including the TWS earphones and Hearing Test APP, provided stable tone playback and controlled test procedures. The hearing thresholds measured using the experimental setup did not significantly differ from the standard hearing thresholds, validating the consistency of the experimental hearing test equipment (TWS earphone playback and Hearing Test APP) with standard audiometric equipment through biological calibration. These findings suggest that the TWS earphones, in conjunction with the Hearing Test APP, can provide pure-tone outputs comparable to those of standard audiometric insert earphones, thereby demonstrating the feasibility of this experimental setup for hearing tests.

### B. Electroacoustic Analysis and Interpretation

Electroacoustic results showed that optimized ANC technology under noisy conditions results in a higher pure-tone SNR than when using generic ANC. However, some discrepancies in the noise cancellation performance of the left and right earphones at 1 kHz when optimizing the ANC. This difference may be attributed to the limitations of manually adjusting the filter coefficients because the optimizing filter design process is manual. The optimization of both earphones considered the center frequency, Q factor, and gain to match the target frequency-response curve for optimized noise reduction. The complexity of the target frequency response curve may be the reason for the differences in noise cancellation performance between the left and right earphones when optimizing the ANC. ANC technology primarily relies on the principles of acoustic superposition and destructive interference to cancel out noise and typically performs better at canceling low-frequency noise [32], [46]. However, various factors can affect the ANC performance. By manually adjusting the filter parameters [46] that match the frequency response to optimize the ANC, the characteristics of the controller can be shaped by selecting appropriate parameters. However, the “waterbed effect” complicates filter design [48], [49]. In addition, the high noise floor observed when the ANC is turned off indicates that the passive noise cancellation capability of the TWS earphone structure is limited, preventing deeper noise cancellation around 0.5 and 1 kHz.

### C. Efficacy and Challenges of Optimized ANC for Hearing Tests

Clinical performance results validated hearing test results at all frequencies using optimized ANC technology were superior to those obtained by generic ANC technology. However, when turning off the ANC at 0.5, 1, 4, and 6 kHz yielded a slightly higher correlation with standard hearing tests than using generic ANC. In addition, combining the electroacoustic results of pure-tone SNRs and the clinical hearing test results, the optimized ANC technology maintained a lower SNR at 0.5 and 1 kHz than SNR at 250 Hz in a 65 dB(A) noise environment and the clinical hearing test results showed the hearing test performance at 0.5 and 1 kHz is inferior to that at 250 Hz as well. A notable finding was that the generic ANC system occasionally produced poorer hearing thresholds than the ANC-off condition, particularly at low frequencies, such as 0.5 and 1 kHz. This counter-intuitive effect is a known artifact of “one-size-fits-all” ANC systems, where a mismatch between the standardized filter and an individual’s ear acoustics can lead to phase errors and inadvertent noise amplification. Furthermore, generic ANC is typically optimized for broadband noise reduction (e.g., across 0–4 kHz) to improve the quality of speech or music. Consequently, its design does not prioritize maximum attenuation at the specific, narrowband pure-tone frequencies used in audiometry. This highlights a fundamental limitation of applying general-purpose technology to a specialized clinical task and underscores the rationale for our proposed method, which is specifically designed to optimize noise reduction at these key audiometric frequencies. Meanwhile, although the overall MAE of all ears was within clinical variability, hearing-impaired ears showed greater measurement variability than normal-hearing ears under the corresponding ANC test conditions. This greater variability may stem from the individualized auditory filtering characteristics of the hearing-impaired ears, such as the increased Equivalent Rectangular Bandwidth (ERB) reported in relevant literature. While a fully personalized ANC system—one that adapts its filters based on an individual’s unique ERB—is acoustically ideal, it faces a significant practical dilemma: implementing such personalization would inherently require a preliminary, time-consuming, individual hearing measurement. This necessity contradicts the core objective of developing a widely accessible, non-clinical, and rapid screening tool. Therefore, advancing ANC technology to quickly and accurately adapt to diverse individual auditory filter characteristics (including those related to HI listeners) without the need for prior clinical measurement remains a critical and indispensable direction for future research. Success in this area will further enhance the reliability and accuracy of self-administered hearing tests for all user populations in noisy environments.

Based on the objective and subjective results with ANC turned off in a noisy environment, we can infer that the passive noise cancellation capability of the TWS earphones used in the experiment was limited. Overall, the ANC system is most effective at reducing low-frequency noise, typically below 2 kHz. Its effectiveness inherently diminished at higher frequencies at which the shorter acoustic wavelengths make it challenging to generate a precise anti-phase signal. Consequently, noise reduction above 2 kHz relies heavily on passive noise cancellation provided by the physical design of the earphones’ features, such as the ear tip seal and housing acoustics. Insufficient isolation in the earphone housing or inadequate sealing between the simple ear tips and ear canal, leading to increased leakage of external noise. If the ANC system components are not of high quality, the system may fail to generate an inverse signal with the same amplitude to effectively counteract external noise, thus reducing the overall ANC performance and negatively affecting hearing test results at specific frequency bands. To address environmental noise fluctuations, the challenge of using ANC technology in hearing tests is to improve the adaptability of the filter to generate noise-canceling signals or to quickly calculate filter coefficients that closely match the target frequency response for different test bandwidths. The overall system performance is a combination of our ANC algorithm and the physical headphone design to significantly mitigate high-frequency issue in applying ANC technology to hearing tests.

## VI. Limitation

It is important to acknowledge that this study evaluated the performance of the proposed ANC system only using stationary pink noise. While this type of noise is frequently used in psychoacoustic research and represents the spectral characteristics of many common household noises, it does not capture the full complexity of real-world environments, particularly the presence of transient and non-stationary sounds. Thus, our results demonstrate the system’s efficacy under controlled, representative conditions. Future research should incorporate a wider variety of noise types, including recorded real-world scenarios and specific transient events, to further validate and enhance the technology’s performance for use in robust self-administered hearing assessments in diverse, everyday settings.

A key limitation of this study arises from the hardware used and corresponding data processing methodology. To develop an accessible and affordable home-based hearing screening tool, this study used consumer-grade wireless earbuds. These devices, while cost-effective, have a more constrained dynamic range than calibrated clinical transducers. Consequently, a measurement floor was established at 25 dB HL—a threshold selected for its clinical relevance in defining the boundary for hearing loss in adults. A direct implication of this design is that the current system cannot resolve different degrees of hearing acuity within the normal range (i.e., below 25 dB HL). Therefore, while the system demonstrates its effectiveness for the intended purpose—screening for clinically significant hearing loss relevant to interventions such as OTC hearing aids—it is not suitable for applications requiring the fine-grained tracking of subtle hearing changes within the normal spectrum. Future research should focus on integrating higher-fidelity hardware or advanced calibration protocols to lower this measurement floor, thereby broadening the system’s clinical and diagnostic applications.

## VII. Conclusion

In this study, we optimized ANC technology based on auditory masking characteristics using the hearing test frequency as the center frequency. Four ANC test conditions were designed to evaluate the subjective and objective performance of ANC. The results of the electroacoustic measurements of the pure-tone SNR and subjective clinical hearing test performance demonstrated consistency across the four ANC test conditions. The proposed optimized ANC technology showed a better correlation between the pure-tone SNR and hearing thresholds than generic ANC or the ANC-off condition. Moreover, the optimized ANC technology outperformed the baseline generic ANC in hearing tests conducted in noisy environments, and the activation of the optimized ANC did not affect the stability of the pure-tone output during the tests. The “optimized ANC” technology proposed in this study, based on auditory masking characteristics of normal hearing, would help users effectively partly overcome environmental noise interference and improve the accuracy of home-based self-administered hearing tests. This would contribute to achieving the core objective of “bringing hearing tests out of the test room.”

## Supplementary Materials

Supplementary Materials

## Conflicts of Interest

The authors declare no conflicts of interest.

## Author Contributions

Hsiu-Lien Cheng and Ying-Hui Lai: designed and performed experiments; Hsiu-Lien Cheng: collected data; Hsiu-Lien Cheng, Po-Hsun Huan, and Ying-Hui Lai: analyzed data and wrote the article; and Wen-Huei Liao: commented on the manuscript revision.
