# Supplementary material for: Optimized Active Noise Cancellation for Hearing Tests Using Auditory Masking Characteristics
Source: IEEE J Transl Eng Health Med. 2025 Nov 6;13:540–51. doi: 10.1109/JTEHM.2025.3629999 (PMC12772985; doi:10.1109/JTEHM.2025.3629999)
Supplement: Supplementary Materials [file supp1-3629999.docx]

Table 1: Frequency Bands of Masking Noise for Hearing Tests (Unit: Hz).

| Center Frequency (Hz) | Critical Bandwidth (Hz) | Low Frequency Limit (Hz) | Upper Frequency Limit (Hz) |
| --- | --- | --- | --- |
| 250 | 105 | 210~223 | 280~297 |
| 500 | 115 | 420~445 | 561~595 |
| 1000 | 160 | 841~891 | 1120~1190 |
| 2000 | 300 | 1680~1780 | 2240~2380 |
| 3000 | 480 | 2520~2670 | 3370~3570 |
| 4000 | 685 | 3360~3560 | 4490~4760 |
| 6000 | 1150 | 5050~5350 | 6730~7140 |
| 8000 | 1700 | 6730~7130 | 8980~9510 |
| Values from ANSI S3.6-2004, p.19 | | | |
