# Supplementary material for: Optimized Active Noise Cancellation for Hearing Tests Using Auditory Masking Characteristics
Source: IEEE J Transl Eng Health Med. 2025 Nov 6;13:540–51. doi: 10.1109/JTEHM.2025.3629999 (PMC12772985; doi:10.1109/JTEHM.2025.3629999)
Supplement: Supplementary Materials [file supp2-3629999.docx]

Table 2: Simple linear regression for ANC scenarios predicting standard audiometric hearing levels at 250 to 8000 Hz.

| **ANC scenarios at 250 Hz** | n | B | SE B | t | p | R^2^ |
| --- | --- | --- | --- | --- | --- | --- |
| S1, ANC on, normal environment | 99 | 0.85 | 0.02 | 37.625 | 0.000 | 0.936 |
| S2, ANC off, noise | 97 | 0.47 | 0.05 | 8.623 | 0.000 | 0.439 |
| S3, generic ANC on, noise | 100 | 0.48 | 0.05 | 9.758 | 0.000 | 0.493 |
| S4, optimized ANC on, noise | 99 | 0.73 | 0.03 | 25.069 | 0.000 | 0.866 |
| **ANC scenarios at 500 Hz** | n | B | SE B | t | p | R^2^ |
| S1, ANC on, normal environment | 100 | 0.84 | 0.02 | 52.314 | 0.000 | 0.965 |
| S2, ANC off, noise | 97 | 0.53 | 0.06 | 9.431 | 0.000 | 0.484 |
| S3, generic ANC on, noise | 97 | 0.46 | 0.05 | 8.789 | 0.000 | 0.448 |
| S4, optimized ANC on, noise | 100 | 0.64 | 0.04 | 14.433 | 0.000 | 0.680 |
| **ANC scenarios at 1000 Hz** | n | B | SE B | t | p | R^2^ |
| S1, ANC on, normal environment | 100 | 0.87 | 0.01 | 61.212 | 0.000 | 0.975 |
| S2, ANC off, noise | 98 | 0.73 | 0.06 | 11.809 | 0.000 | 0.592 |
| S3, generic ANC on, noise | 99 | 0.58 | 0.06 | 10.544 | 0.000 | 0.534 |
| S4, optimized ANC on, noise | 99 | 0.71 | 0.05 | 14.769 | 0.000 | 0.692 |
| **ANC scenarios at 2000 Hz** | n | B | SE B | t | p | R^2^ |
| S1, ANC on, normal environment | 100 | 0.93 | 0.02 | 59.145 | 0.000 | 0.973 |
| S2, ANC off, noise | 98 | 0.71 | 0.05 | 15.477 | 0.000 | 0.714 |
| S3, generic ANC on, noise | 100 | 0.78 | 0.03 | 23.805 | 0.000 | 0.853 |
| S4, optimized ANC on, noise | 100 | 0.99 | 0.03 | 33.837 | 0.000 | 0.921 |
| **ANC scenarios at 3000 Hz** | n | B | SE B | t | p | R^2^ |
| S1, ANC on, normal environment | 100 | 0.91 | 0.02 | 38.982 | 0.000 | 0.939 |
| S2, ANC off, noise | 98 | 0.69 | 0.05 | 13.547 | 0.000 | 0.657 |
| S3, generic ANC on, noise | 100 | 0.91 | 0.05 | 19.888 | 0.000 | 0.801 |
| S4, optimized ANC on, noise | 100 | 0.93 | 0.03 | 31.50 | 0.000 | 0.910 |
| **ANC scenarios at 4000 Hz** | n | B | SE B | t | p | R^2^ |
| S1, ANC on, normal environment | 100 | 0.95 | 0.02 | 43.112 | 0.000 | 0.950 |
| S2, ANC off, noise | 98 | 0.93 | 0.07 | 12.807 | 0.000 | 0.631 |
| S3, generic ANC on, noise | 99 | 0.89 | 0.09 | 9.932 | 0.000 | 0.504 |
| S4, optimized ANC on, noise | 98 | 1.12 | 0.08 | 14.934 | 0.000 | 0.699 |
| **ANC scenarios at 6000 Hz** | n | B | SE B | t | p | R^2^ |
| S1, ANC on, normal environment | 98 | 0.89 | 0.02 | 46.062 | 0.000 | 0.957 |
| S2, ANC off, noise | 98 | 0.89 | 0.05 | 18.123 | 0.000 | 0.774 |
| S3, generic ANC on, noise | 97 | 0.98 | 0.06 | 16.243 | 0.000 | 0.735 |
| S4, optimized ANC on, noise | 99 | 1.06 | 0.07 | 15.591 | 0.000 | 0.715 |
| **ANC scenarios at 8000 Hz** | n | B | SE B | t | p | R^2^ |
| S1, ANC on, normal environment | 98 | 0.82 | 0.02 | 35.431 | 0.000 | 0.929 |
| S2, ANC off, noise | 98 | 0.70 | 0.03 | 20.863 | 0.000 | 0.819 |
| S3, generic ANC on, noise | 97 | 0.77 | 0.03 | 22.357 | 0.000 | 0.840 |
| S4, optimized ANC on, noise | 97 | 0.85 | 0.02 | 35.092 | 0.000 | 0.928 |

B: unstandardized coefficient

SE B: standard error from B

t, p: test statistic and significance level

R^2^: coefficient of determination
